# 3D-printed individualized navigation template versus the fluoroscopic guide to defining the femoral tunnel for medial patellofemoral ligament reconstruction: A retrospective study

**DOI:** 10.1097/MD.0000000000032729

**Published:** 2023-01-27

**Authors:** Wenhao Zhang, Limin Mou, Shiping Zhang, Wei Liu, Aimaiti Remila, Mingzhan Han, Wenyuan Xiang, Rui Fang

**Affiliations:** a Xinjiang Medical University, Urumqi, Xinjiang Province, China; b Department of Orthopedics, The Fourth Affiliated Hospital of Xinjiang Medical University, Urumqi, Xinjiang Province, China; c Emergency Trauma Surgery, Yichang Central Hospital, affiliated with Three Gorges University, Yicang, Hubei Province.

**Keywords:** MPFL reconstruction, patellar dislocation, patellofemoral track, printing, Schöttle point, three-dimensional

## Abstract

During medial patellofemoral ligament (MPFL) reconstruction, fluoroscopic determination of the femoral tunnel point is the most common method. However, there is a decrease in tunnel position accuracy due to rotation of the femur during fluoroscopy, as well as the damage to the operator from multiple fluoroscopies, whereas the 3D-printed individualized navigation template is not affected by this factor. This study focuses on the accuracy and early clinical efficacy of 2 different ways to determine the femoral tunnel (Schöttle point) for double-bundle isometric MPFL reconstruction. This is a retrospective study, conducted between 2016 and 2019, in which 60 patients with recurrent patellar dislocation were divided into 2 groups: 30 with MPFL reconstruction at the Schöttle point determined by 3D-printed individualized navigation template (group A) and 30 with MPFL reconstruction at the Schöttle point determined by fluoroscopic guidance (group B). The changes in patella congruence angle and patella tilt angle before and after surgery were assessed using computed tomography scans of the knee, knee function was assessed using the Kujala knee score and the international knee documentation committee (IKDC) score, and the 2 approaches were compared for the intraoperative establishment of the femoral tunnel position at a distance from Schöttle point. At a minimum of 3 years follow-up, patella tilt angle and patella congruence angle returned to normal levels and were statistically different from the preoperative range, with no significant differences between the 2 groups at the same period, and Kujala and IKDC scores of knee function were significantly improved in both groups after surgery. The mean Kujala and IKDC scores were statistically different between groups A and B at 3 and 6 months postoperatively. No statistically significant differences were seen between the 2 groups at the final follow-up. Both femoral tunnel localization approaches for double-bundle isometric MPFL reconstruction resulted in good knee function. At no < 3 years of follow-up, the use of a 3D-printed individualized navigation template did result in more accurate isometric points and higher knee function scores in the early postoperative period.

## 1. Introduction

Lateral patellar dislocation is a common acute knee injury condition that usually occurs in active pediatric and adolescent patients, with an incidence of patellar dislocation of 77 cases per 1,00,000 pediatric patients.^[1,2]^ Recurrence rates after initial dislocation are as high as 70%.^[3]^ 96% of tears of the medial patellofemoral ligament (MPFL) can occur in initial dislocation,^[4]^ and the MPFL, the primary medial soft tissue restrictive device in limiting lateral patellar displacement, has been recognized as a critical step in the treatment of patients with recurrent patellar dislocation ^[5]^ and provides good outcomes for patellar stability and quality of life.^[3]^ During MPFL reconstruction, anatomical MPFL reconstruction is essential to reestablish graft isometricity and functionality, in which the determination of the femoral tunnel is crucial, and an inappropriate femoral tunnel can lead to patellofemoral imbalance or postoperative re-dislocation and end-stage patellofemoral arthritis, most commonly used in clinical work to obtain the Schöttle point using standard lateral fluoroscopy of the knee, as described by Schöttle^[6]^ et al in 2007. However, intraoperative acquiring standard lateral fluoroscopy is not easy and also concluded that aberration from true lateral of as little as 5° will significantly increase MPFL femoral tunnel malposition.^[7,8]^

Duren^[8]^ et al used prototype drill-guide for patellar tunnel determination, and Liu^[9]^ et al found high localization accuracy and short time by using 3D-printed individualized navigation template for anterior cruciate ligament reconstruction. Kong^[10]^ et al found that 3D printing technique assisted technique shortened operative time reduced blood loss and intraoperative fluoroscopy in the treatment of distal radius intra-articular fractures in patients with K-wire fixation compared to non-assisted technique and concluded that the 3D printing technique is safe and effective in the treatment of distal radius fractures. Wang^[11]^ concluded that the use of a 3D-printed composite guide plate for atlantoaxial pedicle screw fixation to treat atlantoaxial dislocation was superior to the conventional fixation group in terms of screw fixation accuracy, operative time, a number of fluoroscopies, operative time, and intraoperative bleeding. Zhang^[12]^ et al found that the 3D-printed integral customized acetabular prosthesis matched precisely with the reamed acetabulum. The rotation center was restored and the bone defect was exactly reconstructed. There were no signs of prosthetic loosening at the 12-month follow-up. The Harris score gradually improved during the follow-up period. In a case report, the 3D printing technology can be applied to clarify the relationship between blood vessels and bone around the implant to minimize injury to important structures during implantation.^[13]^

Based on our clinical experience, during MPFL reconstruction, the isometric point of the femoral tunnel (Schöttle point) varies due to individual development of the femur, so we verified that the use of 3D-printed individualized navigation template can solve some of the effects of individual differences, and at the same time can reduce the influence of the internal and external rotation of the femur on the positioning of the femoral tunnel during fluoroscopy. This study hypothesized that intraoperative use of the 3D-printed individualized navigation template technique to determine the Schöttle point for MPFL reconstruction compared with the fluoroscopic guidance technique would result in more accurate isometric points and better knee function in the early stages.

## 2. Materials and methods

Sixty patients with recurrent patellar dislocation who satisfied the inclusion and exclusion criteria for MPFL reconstruction between 2016 and 2019 at the Department of Joint Surgery. The sample size of this study was 60 cases, 30 cases in each of the 2 groups. Inclusion criteria: patients aged 18 to 45 years (2) more than 2 dislocations after the first dislocation; painful patellar instability symptoms or subluxation; positive patellar apprehension test;^[14]^ excessive patellar tilt angle on computed tomography (CT) and MPFL tear on magnetic resonance imaging. Exclusion criteria: age < 18 years or more than 45 years; bony structural abnormalities: Insall-Salvati index ≥ 1.2,^[15]^ tibial tuberosity-trochlear groove distance of ≥ 20 mm,^[16]^ Severe Trochlear Dysplasia (Dejour type B, C, or D),^[17]^ Femoral anteversion angle ≥ 25°,^[18]^ and previous surgery on the affected knee, all performed by a surgically experienced chief surgeon. All knee function scores and imaging measurements, assessments and follow-up, and documentation were performed by a single blinded observer. The precision of our measurements was 0.1 mm and 0.1°. Preoperative approval was obtained from the local ethics committee for the 60 participants included, including informing patients of the surgical procedure, and possible postoperative complications signing an informed consent form, and ethical approval number (LFYLLSC20160104-03).

### 2.1. Preparation of the individualized navigation templates

**Figure 1.**
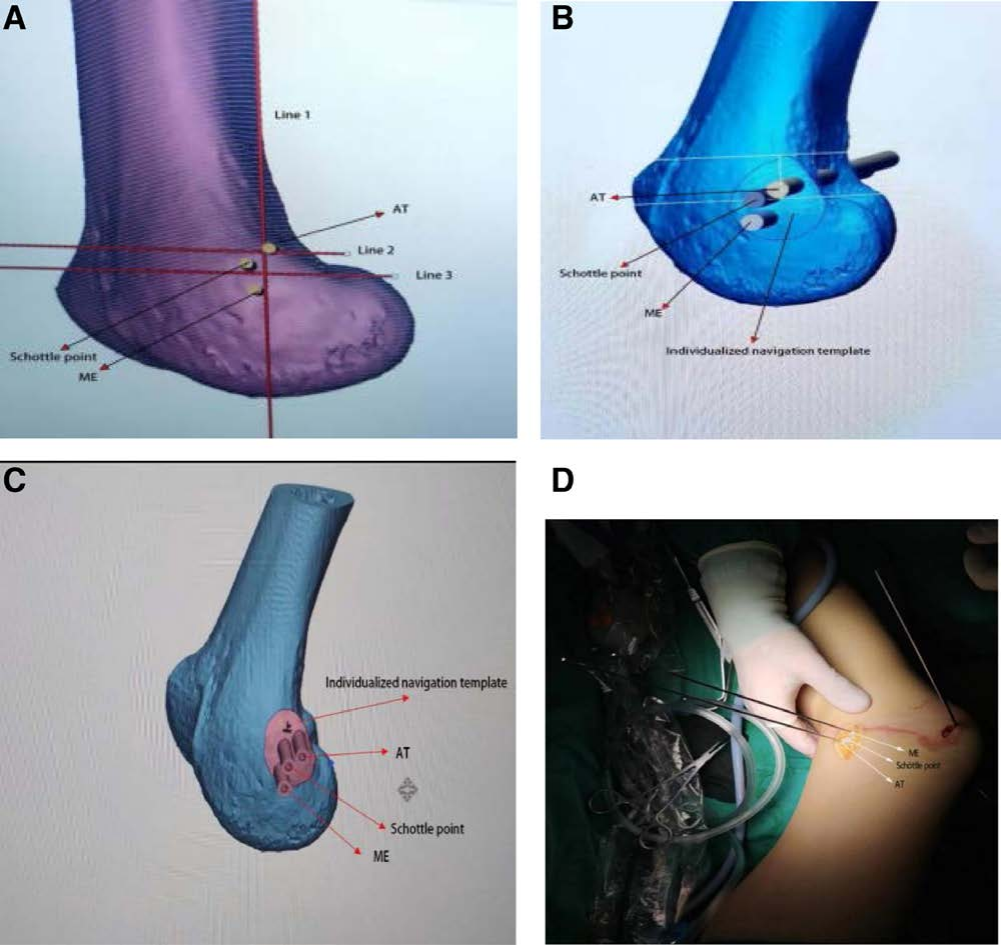
a Preliminary determination of Schöttle point using Mimics Research. (b) Identify individualized navigation template and bony landmark locations in 3d-matic. (c) Final production of individualized navigation template. (d) 3D-printed individualized navigation template was used intraoperatively to determine the location of the femoral tunnel.

For the 3D-printed individualized navigation template group, a CT scan + 3D reconstruction of the affected knee in extension was used, and the CT data were imported into the hard disk in Digital Imaging and Communications in Medicine (DICOM) format. The data were imported into Mimics Research 19.0 (Materialise, Belgium) for image processing to generate a 3D image of the distal femur, adjusting the 3D image of the distal femur to a standard lateral position. Schöttle^[6]^ proposed the use of 3 lines, as Figure 1a. line 1 was an extension of the posterior femoral cortex, line 2 intersected the contact of the posterior femoral condyle with the posterior cortex, and line 3 intersected the posterior point of the Blumensaat line. Then the Schöttle point^[6]^ of the femoral medial patellofemoral ligament was determined by lines 1, 2, 3.

The medial epicondyle (ME) and the adductor tubercle (AT), and the Schöttle point were marked on the medial side of the 3D image by 3 parallel columns of 6 mm diameter, and the file was saved. The file in STL (STereo Lithography) format was exported and imported into Materialise Magics 21.0 (Materialise, Belgium). Three 6 mm diameter horizontal circular columns are replicated in 3 equally positioned 2 mm diameter columns, and an individualized navigation template is created on the bone surface where the columns intersect the medial femoral epicondyle, with a wall thickness of 2 mm, so that the guide plate fits the bone surface completely, as Figure 1b, using 3 6 mm diameter parallel columns minus the corresponding 2 mm diameter columns. The blank portion of the remaining cylinders is used to determine the AT and ME and Schöttle point positions for the 3 intra-operative kirschner wire, as Figure 1c, to finalize the 3D-printed individualized navigation template, which were made by a 3D printer (Creality, Shenzhen, China). The print guides are made of polylactic acid material (a nontoxic material that is safe for clinical work) to print the models, and the high compressive strength is 97.2–98.7 MPa.^[19]^

### 2.2. Technique for MPFL reconstruction

All patients were treated with spinal anesthesia with the patient in the supine position, and a tourniquet was applied to the proximal femur with the pressure adjusted to 50 Kpa. A routine diagnostic arthroscopy of the affected knee was performed with anterolateral and anteromedial approaches to the knee to explore the patellofemoral joint tracks, patellar dislocation, cartilage damage, meniscal damage, and other conditions using arthroscopic Shaver (Stryker, American) to clear the free synovial membrane. The autologous peroneus longus tendon was taken and prepared for knitting, and the skin was incised longitudinally in the mid-superior 1/3 of the medial border of the patella, a parallel patellar tunnel was drilled with a 4.5 mm cannulated reamer, and the graft was placed in the patellar tunnel and fixed using a Suture Anchor (Johnson & Johnson, American) fixation screwed into the patellar tunnel, and the graft was placed in the graft was retracted between the first and second layers of the knee capsule to the femoral side.

In group A: 1 surgeon initially determined the location of the femoral tunnel by touching the medial epicondyle, made a longitudinal incision of approximately 2.0 cm along the long axis of the femoral stem, separated the subcutaneous tissues and muscles, fully exposed the bony landmark ME and AT and affixed a 3D-printed individualized navigation template to the femoral surface. Two 1.8 mm Kristen pins were passed through the fixation holes on the guide plate and fixed to the ME and AT, as shown in Figure 1d, then 1 1.8 mm Kristen pin was used to determine the Schöttle point and a 25 mm deep tunnel was drilled through the Kristen pin using an 8 mm diameter reamer. The tail end of the graft is drawn to the lateral femoral tunnel using a wire, and the appropriate patellofemoral relationship is determined arthroscopically while the knee is flexed and extended, at which time the tension of the graft is maintained and an interference screw is used to fix it to the femoral tunnel.

**Figure 2.**
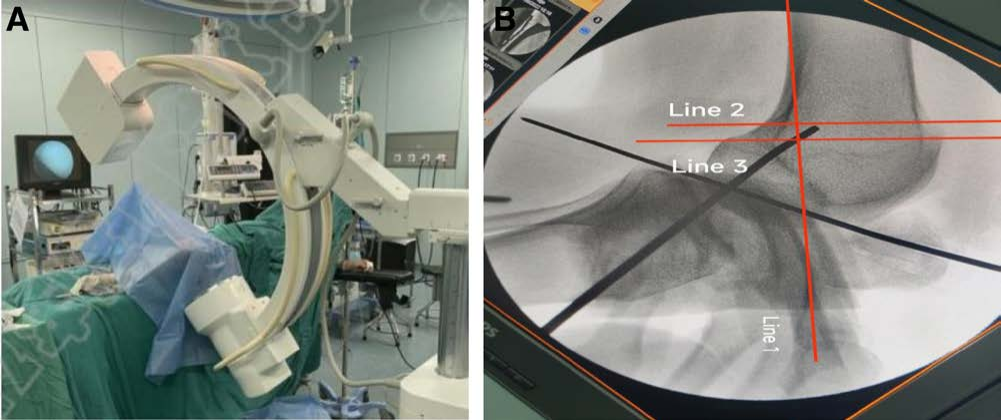
(a) Intraoperative determination of femoral insertion by fluoroscopy. (b) Determine the femoral stop position through 3 lines after fluoroscopy.

In group B: The preliminary assessment of the femoral tunnel location was the same as in group A by 1 surgeon. After exposing the bony landmark ME and AT, the Schöttle point was determined using a 1.8 mm Kerschner pin by choosing the standard lateral fluoroscopy of the knee under the fluoroscopy machine,^[6]^ Figure 2a, b, and a 25 mm deep tunnel was drilled on the Kerschner pin using an 8 mm diameter reamer, and the end of the graft was fixed to the femoral tunnel using a wire The end of the graft was drawn to the lateral side of the femoral tunnel, and the patellofemoral joint was determined arthroscopically by flexion and extension of the knee while maintaining appropriate tension, and then fixed to the femoral tunnel using an interference screw.

### 2.3. Postoperative rehabilitation and assessment

Postoperatively, the brace can be adjusted to 0° fixation, the drainage tubing is removed on the 2nd day, and the patient is instructed to perform early exercises of straight leg raising to restore muscle strength, and wear the brace to move on the ground with the aid of crutches after 1 week postoperatively under the guidance of the rehabilitation teacher, and actively flex the knee within 30° in bed, and adjust the angle of the brace according to the patient’s flexion and extension in 2 weeks postoperatively, and perform training of knee flexion 0 to 60°, increasing by 10 to 20° every week, and reach 90° of knee flexion around 3 to 5 weeks postoperatively The brace can be removed at rest. The brace was removed 2 to 3 months after surgery for walking and jogging, and normal sports activities were resumed after 4 months. In the rehabilitation process of both groups of patients, particular attention should be drawn to the following: firstly a professional rehabilitation physician is essential for the postoperative rehabilitation program, secondly, it is necessary to avoid violent activities when the affected limbs are on the ground, and thirdly pay attention to the psychological guidance and guidance of the patients and encourage them to perform appropriate rehabilitation activities.

Using the Kujala score^[20]^ and international knee documentation committee (IKDC) score^[21]^ to assess knee function preoperatively, at 3 months postoperatively, at 6 months, and at the final follow-up, each patient underwent frontal and lateral fluoroscopy of the knee with CT scans, to determine patella tilt angle (PTA)^[22]^ and patella congruence angle (PCA),^[23]^ both PTA and PCA measured 1 to 3 preoperatively and 7 days postoperatively were used to assess the patellofemoral joint relationship and the distance between the femoral tunnel and Schöttle point in both groups.

### 2.4. Statistical analyses

Statistical analysis was performed using SPSS 26.0 software (IBM, Armonk, NY). The Kolmogorov-Smirnov test was used to test the normality of the variances, and measurement data were expressed as mean ± standard deviation (X̄ ± S), Comparisons between the 2 groups were made using the *t* test for 2 independent samples; the count data were expressed as percentages (%), and comparisons between the 2 groups were made using the χ^2^ test, with a test level of = 0.05, with *P* < .05 indicating a statistically significant difference between the 2 groups.

## 3. Results

There were 60 patients in both groups, but the differences were not statistically significant (*P* > .05), Table 1. When comparing age, gender, right and left knee, BMI, number of preoperative dislocations and follow-up time in the 2 groups, respectively.

**Table 1:** Demographic characteristics of the patient.

Variable	Group A (n = 30)	Group B (n = 30)	*P* value
Males/females	14/16	17/13	.597
Age (yr)	24.37 ± 6.83	22.47 ± 5.50	.24
BMI (body mass index)	27.25 ± 3.08	27.13 ± 4.47	.902
Left leg/right leg	14/16	17/13	.571
Number of dislocations	2.97 ± 0.93	2.90 ± 0.92	.781
follow-up (mo)	43.23 ± 5.56	42.13 ± 4.51	.404

**Figure 3.**
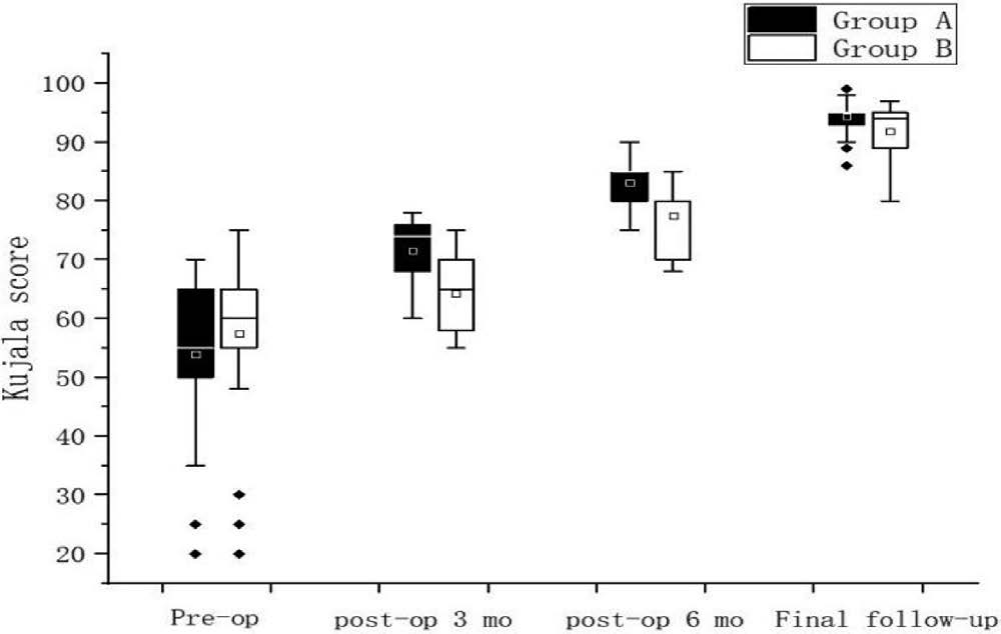
Comparison of Kujala scores at different time points.

**Figure 4.**
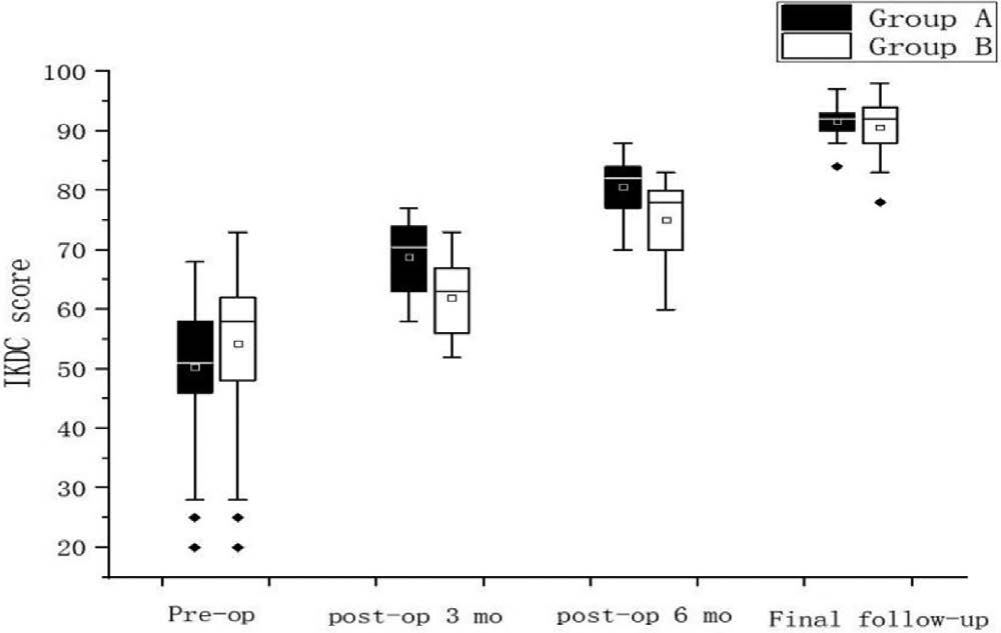
Comparison of IKDC scores at different time points. IKDC = international knee documentation committee.

**Figure 5.**
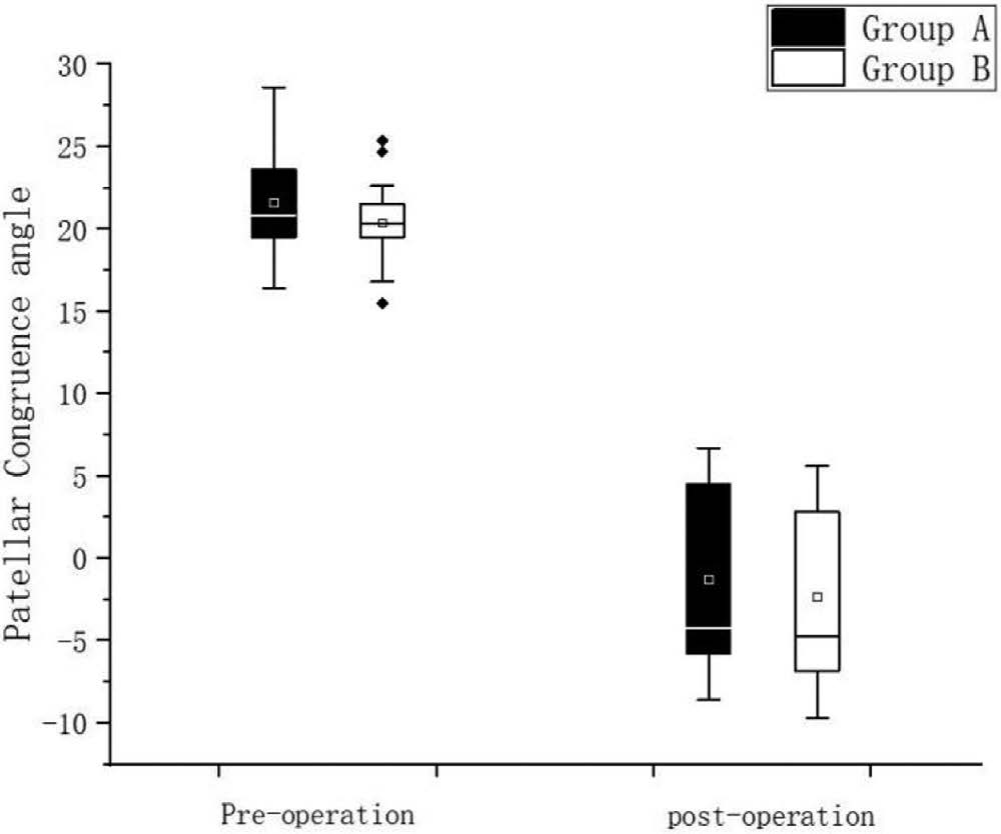
Comparison of preoperative and postoperative PCA. PCA = patella congruence angle.

**Figure 6.**
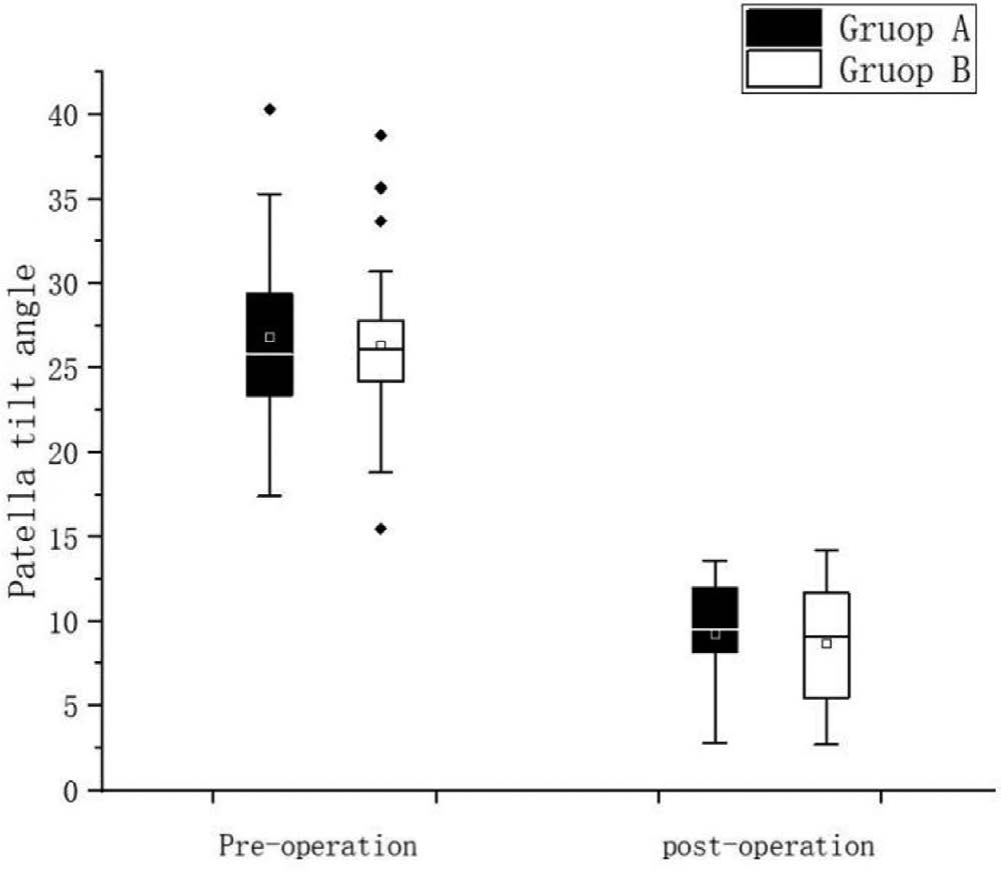
Comparison of preoperative and postoperative PTA. PTA = patella tilt angle.

**Figure 7.**
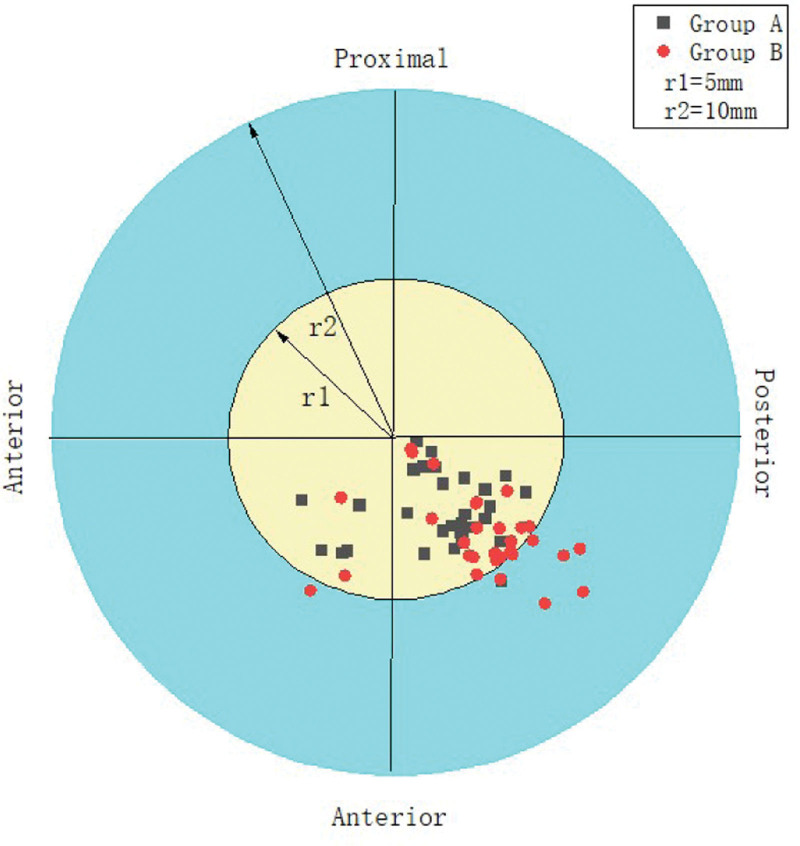
Comparison of femoral isometric point positions between the 2 groups.

The PCA, PTA, Kujala score and IKDC score recovered to normal knee level in both groups after surgery, and the differences were statistically significant (*P* < .05) compared with preoperative levels, Table 2; Kujala score and IKDC were higher in Group A than in Group B in 3 and 6 months after surgery, and the difference was statistically significant (*P* < .05), but no significant difference was seen between the 2 groups at the final follow-up, Figure 3, Figure 4, There was no statistically significant difference in the postoperative PCA and PTA between the 2 groups of patients (*P* > .05) Figure 5, Figure 6. The isometric point distances of the 2 groups were: 3.10 ± 1.06 mm and 4.27 ± 1.56, respectively, and the isometric point distance of Group A was more accurate than Group B, and the difference was statistically significant (*P* < .05) Fig 7.

**Table 2:** Comparisons of preoperative patella associated measurements and knee function between the 2 groups.

	Group A (n = 30)	Group B (n = 30)	*P* value
PCA			
Pre-operation	21.63 ± 3.02	20.39 ± 2.08	.07
Post-operation	(−) 1.27 ± 5.55	(−) 2.34 ± 5.31	.452
*P* value	.000	.000	
PTA			
Pre-operation	26.86 ± 4.85	26.33 ± 5.15	.685
Post-operation	9.25 ± 3.18	8.73 ± 3.36	.541
*P* value	.000	.000	
Isometric point	3.10 ± 1.06	4.27 ± 1.56	.000

PCA = patella congruence angle, PTA = patella tilt angle.

### 3.1. Complications

All patients had primary healing of the incision, and no infection or blood embolism was seen. No re-dislocation was seen in either group after surgery, and 3 cases of pain at the medial edge of the patella occurred in each of the 2 groups when the temperature became cold, and 1 case of knee flexion deficit occurred in Group B 3 months after surgery, and knee release under anesthesia was given.

## 4. Discussion

The most important finding of this study was that MPFL reconstruction using a 3D-printed individualized navigation template to determine femoral isometric points resulted in more accurate femoral tunnel localization and higher subjective Kujala and IKDC knee function scores in the early postoperative period, including 3 and 6 months, compared to fluoroscopic guidance.

The footprint of reconstructed MPFL include the femoral footprint and patellar footprint, because the patellar footprint has less influence on postoperative knee function than the femoral footprint, researchers have focused more on the femoral footprint,^[24,25]^ and in clinical work, non-isometric reconstruction is more common than expected, most of which may be related to the surgeon’s operation, and the way to judge the isometric of the graft is controversial, the first method is Smirk^[26]^ considered that a change in graft length of < 5 mm within 0 to 90° is considered isometric reconstruction. Another commonly used method to determine isometric is the postoperative measurement of the length of the femoral tunnel from the Schöttle point, some researchers consider the femoral tunnel to be < 5 mm from the isometric point, for isometric reconstruction,^[27,28]^ while others consider the femoral tunnel to be < 7 mm from the isometric point,^[29]^ and even McCarthy et al^[30]^ consider the femoral tunnel to be < 9 mm from the isometric point to be reasonable, if the graft femoral tunnel is only 5 mm proximal or distal to the isometric point, this would result in a 12 mm change in graft length, resulting in a non-isometric graft.^[31]^

Therefore, the location of the femoral footprint is crucial in isometric reconstruction, and Thaunat^[32]^ et al concluded that placing the femoral tunnel location too close to the patellar footprint can lead to a significant increase in graft tension during knee flexion, resulting in early medial patellar edge pain; conversely, placing the femoral tunnel location too far away can result in insufficient graft tension to create medial restraint. In this study, the distance between the femoral stop and the isometric point was determined to be 3.42 ± 0.81 using a 3D-printed individualized navigation template and 4.67 ± 1.24 in the fluoroscopic, and the distance in the 3D-printed individualized navigation template group was < that in the fluoroscopic group, so we concluded that more accurate isometric femoral positioning points could be obtained using 3D printing technology, and the 3D-printed individualized navigation template was not only inexpensive but also accurate, reducing intraoperative time to determine the femoral tunnel, and all postoperative incisions healed in 1 stage. No infected sinus tracts were observed; during the follow-up, no redislocation and no knee flexion and extension disorders were observed, so it can be safely applied to ligament reconstruction.

Schöttle^[6]^ uses fluoroscopy to determine the Schöttle point is a common clinical approach, Ziegler^[33]^ and Wijdick^[34]^ proposed a point similar to the Schöttle point based on Schöttle and also verified its validity, but recently it is also controversial because the intraoperative acquisition of a standard lateral position is not easy. Stephen^[31]^ proposed 2 ways to determine the stop point, the first using the midpoint of 2 bony landmarks, ME and AT, to determine the femoral stop point, and the second by fluoroscopy, by determining the size of the anterior-posterior diameter of the medial femoral condyle as 100%, the MPFL femoral tunnel localization point should be 60% from the anterior end, 40% from the posterior end and 50% from the distal end; on the other hand, Ziegler et al^[33]^ considered that the femoral tunnel localization point should be 11.4 mm from ME and 7.8 mm from AT, Wijdicks et al^[34]^ considered that the femoral tunnel localization point should be 15.9 mm from ME and 8.9 mm from AT; Chen^[24]^ et al used the Saddle, AT and Medial gastrocnemius tubercle (MGT) surrounded by 3 points as the Sulcus center as the femoral stop and compared with Schöttle’s method, the femoral stop distance from the ischial point averaged 5.9 mm and 6.2 mm, respectively, but the potential variability of the ME position and the wide and flat shape of the ME apex can sometimes have an impact on the determination of the femoral stop by bony landmarks.

3D-printed individualized navigation template may reduce tunnel positioning errors by less experienced surgeons, improve growth plate retention in skeletally immature patients, reduce radiation exposure by not using intraoperative fluoroscopy.

With the increasing complexity of the surgery and surgical decision-making, 3D printing technology has emerged as a new surgical modality with the potential and convenience to make a huge impact in the field of surgery. The main ones include: pre-operative planning; 3D implants; 3Dpatient-specific instrumentation (PSI), pre-operative planning allows surgeons to visualize relevant anatomical structures and helps to perform complex surgery,^[9]^ 3D implants can be used for direct replacement of large defects after tumor resection and to aid reconstruction in limb-preserving surgery,^[35]^ and intraoperative use of PSI can be used to a large extent for more accurate placement of internal fixations, especially in the presence of abnormal anatomy and deformities. Duren^[8]^ et al used a prototype drill guide to perform patellar tunnel determination in MPFL reconstruction, which improved accuracy and reduced time compared to the conventional approach, and Liu^[9]^ et al also verified the effectiveness of this technique by using 3D-printed individualized navigation template to perform ACL reconstruction, which is consistent with the results of this study.

## 5. Limitations

The study was subject to a small sample size, and there may be partial inclusion of biased chairs, and the sample size should be increased in future studies; second, the follow-up period was short, and the follow-up period should be extended for the included patients in future work to understand the long-term prognosis of the patients; third, due to the effect of COVID-19 virus, some patients were followed up by sending emails to fill in questionnaires to understand the recovery of knee function, and in face-to-face, follow-up was chosen as much as possible.

## 6. Conclusion

Both femoral tunnel localization approaches for double-bundle isometric MPFL reconstruction resulted in good knee function, and at no < 3 years of follow-up, the use of 3D-printed individualized navigation template did result in more accurate isometric points and higher knee function scores in the early postoperative period.

## Author contributions

**Conceptualization:** Wenhao Zhang, Shiping Zhang, Remila·Aimaiti.

**Data curation:** Wenhao Zhang, Limin Mou.

**Formal analysis:** Wenhao Zhang, Remila·Aimaiti, Mingzhan Han.

**Funding acquisition:** Limin Mou, Wei Liu.

**Investigation:** Wenyuan Xiang.

**Methodology:** Wenhao Zhang, Limin Mou.

**Project administration:** Wenhao Zhang, Wei Liu, Wenyuan Xiang.

**Resources:** Wenhao Zhang, Wei Liu, Remila·Aimaiti.

**Software:** Remila·Aimaiti, Mingzhan Han.

**Supervision:** Remila·Aimaiti, Mingzhan Han.

**Visualization:** Shiping Zhang, Mingzhan Han, Rui Fang.

**Writing – original draft:** Shiping Zhang, Rui Fang.

**Writing – review & editing:** Wenyuan Xiang, Rui Fang.
